# Supplementary material for: An open-source framework for large-scale, flexible evaluation of biomedical text mining systems
Source: J Biomed Discov Collab. 2008 Jan 29;3:1. doi: 10.1186/1747-5333-3-1 (PMC2276192; doi:10.1186/1747-5333-3-1)
Supplement: Additional file 1 — Evaluation platform source code. The additional file contains the software evaluation framework discussed in this paper. The most current release can be downloaded from SourceForge.net [1]. See the accompanying README file for instructions on installation and use. Also included in the distribution is a collection of UIMA wrappers for some commonly used BioNLP tools and annotated corpora. [file 1747-5333-3-1-S1.zip › bionlp_uima_repository-1.0.1/docs/api/edu/uchsc/ccp/util/nlp/mention/ClassMention.html]

ClassMention (Center for Computational Pharmacology Util/UIMA API)


|  |  |  |
| --- | --- | --- |
| **Overview** \| **Package** \| **Class** \| **Tree** \| **Deprecated** \| **Index** \| **Help** | |  |
| PREV CLASS   **NEXT CLASS** | **FRAMES**    **NO FRAMES**     **All Classes** |  |
| SUMMARY: NESTED \| FIELD \| CONSTR \| METHOD | DETAIL: FIELD \| CONSTR \| METHOD |  |


---


## edu.uchsc.ccp.util.nlp.mention Class ClassMention

```
java.lang.Object
  edu.uchsc.ccp.util.nlp.mention.Mention
      edu.uchsc.ccp.util.nlp.mention.ClassMention
```

**All Implemented Interfaces:**: Comparable

---

``` public class ClassMention extends Mention ```

---

**Constructor Summary**

`ClassMention()`
  
          **Deprecated.**

`ClassMention(String mentionName)`


| **Method Summary** | |
| --- | --- |
| `void` | `addComplexSlotMention(ComplexSlotMention complexSlotMention)` |
| `void` | `addSlotMention(SlotMention slotMention)` |
| `void` | `addTextAnnotation(TextAnnotation textAnnotation)` |
| `int` | `compareTo(Object o)`             The default compareTo uses the IdenticalClassMentionComparator() |
| `boolean` | `equals(Object classMentionToEquate)`             The default equals() method uses the IdenticalClassMentionComparator. |
| `ComplexSlotMention` | `getComplexSlotMentionByName(String complexSlotMentionName)`             **Deprecated.** |
| `List<ComplexSlotMention>` | `getComplexSlotMentions()` |
| `List<ComplexSlotMention>` | `getComplexSlotMentionsByName(String complexSlotMentionName)`             Return all complex slot mentions for a given name. |
| `String` | `getDocumentLevelSingleLineRepresentation()`             similar to getSingleLineRepresentation, however this is a document-level representation, so no meta data are included (span info, covered text, etc.) |
| `SlotMention` | `getFirstSlotMention()`             Return the first slot mention |
| `String` | `getHashKey()` |
| `String` | `getSingleLineRepresentation()`             outputs a single line representation of this class mention. |
| `SlotMention` | `getSlotMentionByName(String slotMentionName)`             **Deprecated.** |
| `List<String>` | `getSlotMentionNames()`             Returns the unique names of all slotmentions |
| `List<SlotMention>` | `getSlotMentions()` |
| `List<SlotMention>` | `getSlotMentionsByName(String slotMentionName)`             Return all slot mentions for a given name. |
| `List<TextAnnotation>` | `getTextAnnotations()` |
| `static void` | `printMention(ClassMention cm, PrintStream ps, int indentLevel)` |
| `void` | `setComplexSlotMentions(List<ComplexSlotMention> complexSlotMentions)` |
| `void` | `setSlotMentions(List<SlotMention> slotMentions)` |
| `void` | `setTextAnnotation(List<TextAnnotation> textAnnotations)` |

**Methods inherited from class edu.uchsc.ccp.util.nlp.mention.Mention**

`getMentionName, setMentionName`

**Methods inherited from class java.lang.Object**

`clone, finalize, getClass, hashCode, notify, notifyAll, toString, wait, wait, wait`

**Constructor Detail**

### ClassMention

```
@Deprecated
public ClassMention()
```

:   **Deprecated.**

---


### ClassMention

```
public ClassMention(String mentionName)
```


**Method Detail**

### getTextAnnotations

```
public List<TextAnnotation> getTextAnnotations()
```

---


### setTextAnnotation

```
public void setTextAnnotation(List<TextAnnotation> textAnnotations)
```

---


### addTextAnnotation

```
public void addTextAnnotation(TextAnnotation textAnnotation)
```

---


### getComplexSlotMentions

```
public List<ComplexSlotMention> getComplexSlotMentions()
```

---


### setComplexSlotMentions

```
public void setComplexSlotMentions(List<ComplexSlotMention> complexSlotMentions)
```

---


### getSlotMentions

```
public List<SlotMention> getSlotMentions()
```

---


### setSlotMentions

```
public void setSlotMentions(List<SlotMention> slotMentions)
```

---


### addComplexSlotMention

```
public void addComplexSlotMention(ComplexSlotMention complexSlotMention)
```

---


### addSlotMention

```
public void addSlotMention(SlotMention slotMention)
```

---


### getSlotMentionNames

```
public List<String> getSlotMentionNames()
```

:   Returns the unique names of all slotmentions

    :   **Returns:**

---


### getSlotMentionByName

```
@Deprecated
public SlotMention getSlotMentionByName(String slotMentionName)
```

:   **Deprecated.**

---


### getSlotMentionsByName

```
public List<SlotMention> getSlotMentionsByName(String slotMentionName)
```

:   Return all slot mentions for a given name. If there are no slot mentions for a given name, null is returned.

    :   **Parameters:**: `slotMentionName` - **Returns:**

---


### getComplexSlotMentionByName

```
@Deprecated
public ComplexSlotMention getComplexSlotMentionByName(String complexSlotMentionName)
```

:   **Deprecated.**

---


### getComplexSlotMentionsByName

```
public List<ComplexSlotMention> getComplexSlotMentionsByName(String complexSlotMentionName)
```

:   Return all complex slot mentions for a given name. If there are no complex slot mentions for a given name, null is returned.

    :   **Parameters:**: `complexSlotMentionName` - **Returns:**

---


### getFirstSlotMention

```
public SlotMention getFirstSlotMention()
```

:   Return the first slot mention

    :   **Returns:**

---


### getSingleLineRepresentation

```
public String getSingleLineRepresentation()
```

:   outputs a single line representation of this class mention. Slots are alphabetized before output so that they are always in the same order

    :   **Overrides:**: `getSingleLineRepresentation` in class `Mention`
    :   **Returns:**

---


### getDocumentLevelSingleLineRepresentation

```
public String getDocumentLevelSingleLineRepresentation()
```

:   similar to getSingleLineRepresentation, however this is a document-level representation, so no meta data are included (span info, covered text, etc.)

    :   **Overrides:**: `getDocumentLevelSingleLineRepresentation` in class `Mention`
    :   **Returns:**

---


### printMention

```
public static void printMention(ClassMention cm,
                                PrintStream ps,
                                int indentLevel)
```

---


### getHashKey

```
public String getHashKey()
```

---


### compareTo

```
public int compareTo(Object o)
```

:   The default compareTo uses the IdenticalClassMentionComparator()

    :   **Specified by:**: `compareTo` in interface `Comparable` **Overrides:**: `compareTo` in class `Mention`

---


### equals

```
public boolean equals(Object classMentionToEquate)
```

:   The default equals() method uses the IdenticalClassMentionComparator.

    :   **Overrides:**: `equals` in class `Object`


---


|  |  |  |
| --- | --- | --- |
| **Overview** \| **Package** \| **Class** \| **Tree** \| **Deprecated** \| **Index** \| **Help** | |  |
| PREV CLASS   **NEXT CLASS** | **FRAMES**    **NO FRAMES**     **All Classes** |  |
| SUMMARY: NESTED \| FIELD \| CONSTR \| METHOD | DETAIL: FIELD \| CONSTR \| METHOD |  |


---
